# Global Analysis of *WRKY* Genes and Their Response to Dehydration and Salt Stress in Soybean

**DOI:** 10.3389/fpls.2016.00009

**Published:** 2016-02-01

**Authors:** Hui Song, Pengfei Wang, Lei Hou, Shuzhen Zhao, Chuanzhi Zhao, Han Xia, Pengcheng Li, Ye Zhang, Xiaotong Bian, Xingjun Wang

**Affiliations:** Biotechnology Research Center, Shandong Academy of Agricultural Sciences, Shandong Provincial Key Laboratory of Crop Genetic Improvement, Ecology and PhysiologyJinan, China

**Keywords:** codon usage bias, dehydration stress, *Glycine max*, salt stress, WRKY protein

## Abstract

WRKY proteins are plant specific transcription factors involved in various developmental and physiological processes, especially in biotic and abiotic stress resistance. Although previous studies suggested that WRKY proteins in soybean (*Glycine max* var. Williams 82) involved in both abiotic and biotic stress responses, the global information of WRKY proteins in the latest version of soybean genome (Wm82.a2v1) and their response to dehydration and salt stress have not been reported. In this study, we identified 176 GmWRKY proteins from soybean Wm82.a2v1 genome. These proteins could be classified into three groups, namely group I (32 proteins), group II (120 proteins), and group III (24 proteins). Our results showed that most *GmWRKY* genes were located on Chromosome 6, while chromosome 11, 12, and 20 contained the least number of this gene family. More *GmWRKY* genes were distributed on the ends of chromosomes to compare with other regions. The *cis*-acting elements analysis suggested that *GmWRKY* genes were transcriptionally regulated upon dehydration and salt stress. RNA-seq data analysis indicated that three *GmWRKY* genes responded negatively to dehydration, and 12 genes positively responded to salt stress at 1, 6, and 12 h, respectively. We confirmed by qRT-PCR that the expression of *GmWRKY47* and *GmWRKY 58* genes was decreased upon dehydration, and the expression of *GmWRKY92, 144* and *165* genes was increased under salt treatment.

## Introduction

WRKY transcription factors were first identified from plant species and were thought to be plant specific (Eulgem et al., 2000; Rushton et al., 2010). However, increasing studies identified WRKY proteins from non-plant species, including *Caenorhabditis elegans, Dictyostelium discoideum, Drosophila melanogaster, Giardia lamblia, Klebsormidium flaccidum*, and *Saccharomyces cerevisiae* (Riechmann et al., 2000; Zhang and Wang, 2005; Rinerson et al., 2015). WRKY proteins were named after a conserved WRKY domain, containing the WRKYGQK heptapeptide, followed by a zinc-finger motif (CX_4−5_CX_22−23_HXH or CX_7_CX_23_HXC; Eulgem et al., 2000; Rushton et al., 2010). Based on the number of WRKY domains and the type of zinc-finger motif, WRKY proteins were classified into three groups (group I–III; Eulgem et al., 2000; Rushton et al., 2010). Group I WRKY proteins contained two WRKY domains and a zinc-finger motif (CX_4−5_CX_22−23_HXH or CX_7_CX_23_HXC). Group II WRKY proteins which could be divided into five subgroups (IIa–IIe), contained a single WRKY domain and a CX_4−5_CX_22−23_HXH zinc-finger motif (Eulgem et al., 2000; Rushton et al., 2010). Group III WRKY proteins had a single WRKY domain and a CX_7_CX_23_HXC zinc-finger motif (Eulgem et al., 2000; Rushton et al., 2010).

To date, genome-wide WRKY analysis has been performed in many plant species including *Arabidopsis thaliana* (Eulgem et al., 2000), *Brachypodium distachyon* (Tripathi et al., 2012; Wen et al., 2014), *Hordeum vulgare* (Liu et al., 2014), *Lotus japonicus* (Song et al., 2014), *Medicago truncatula* (Song and Nan, 2014), *Oryza sativa* (Wu et al., 2005), *Vitis vinifera* (Wang et al., 2014), *Zea mays* (Wei et al., 2012), *Gossypium* (Ding et al., 2015), and *Populus* (He et al., 2012; Jiang et al., 2014). Over the past 15 years, studies demonstrated that WRKY proteins played crucial roles in pathogen defense and insect resistance (Eulgem and Somssich, 2007; Grunewald et al., 2008; Skibbe et al., 2008; Rushton et al., 2010). WRKY proteins were implicated to modulate plant development such as seed development (Luo et al., 2005), trichome morphogenesis (Johnson et al., 2002), senescence (Robatzek and Somssich, 2002), dormancy and germination (Zhang et al., 2004; Zentella et al., 2007; Zou et al., 2008). Recently, studies demonstrated that WRKY proteins were involved in response to abiotic stresses, such as salt, drought, and cold (Wu et al., 2009; Ren et al., 2010; Zou et al., 2010; Jiang et al., 2012; Rushton et al., 2012). WRKY proteins were involved in signal transductions mediated by plant hormones, for example, abscisic acid (ABA) (Rushton et al., 2012). In *Arabidopsis*, WRKY proteins are involved in regulation of ABA-responsive genes, such as *MYB2, DREB1a, DREB2a*, and *RAB18* (Rushton et al., 2012). WRKY proteins could increase drought and salt tolerance in plants. Overexpression of *ZmWRKY33* in *Arabidopsis* could improve salt stress tolerance of the transgenic plants (Li et al., 2013). Overexpression of *OsWRKY11* under the control of HSP101 promoter led to enhanced drought tolerance (Wu et al., 2009). Moreover, overexpression of *TaWRKY10* in tobacco resulted in enhanced drought and salt tolerance (Wang et al., 2013).

Soybean (*Glycine max*), as one of the important protein and oil crop, is planted worldwide. Zhou et al. (2008) identified 64 *GmWRKY* genes before the soybean genome was sequenced, and confirmed that *GmWRKY13, 21*, and *54* genes were involved in abiotic stresses. Bencke-Malato et al. (2014) identified 182 *GmWRKY* genes including 33 pseudogenes using the whole genome sequence information (Wm82.a1.v1; Schmutz et al., 2010). Among the 149 non-pseudogenized *GmWRKY* genes, 72 genes were differentially expressed during fungal infection based on SAGE, RNA-seq and microarray experiments (Bencke-Malato et al., 2014). However, the role of *GmWRKY* gene in dehydration and salt stress are largely unknown. We carried out a comprehensive analysis of *GmWRKY* genes base on the newly released genome Wm82.a2v1, and investigated their response to dehydration and salt stress. Totally, we identified 176 putative *GmWRKY* genes from soybean Wm82.a2v1 genome using bioinformatics approach. The chromosomal location, codon usage bias, *cis*-elements, and gene expression in different tissues and under dehydration and salt stress were analyzed. These results provided new insight into the roles of soybean *WRKY* genes in abiotic stress responses.

## Materials and methods

### Identification and analysis of WRKY genes in soybean

Sequences of the soybean genome (Wm82.a2.v1) were downloaded from Phytozome 10.0 database (http://www.phytozome.org). The Hidden Markov Model (HMM) profile of WRKY domain (PF03106) was downloaded from Pfam protein family database (http://pfam.janelia.org) and was used to survey all soybean proteins by HMMER program (Finn et al., 2011). To verify the reliability of searched results, each protein sequence was checked in Pfam database.

*A. thaliana* WRKY proteins (http://www.arabidopsis.org) were obtained and used for phylogenetic analysis. To categorize GmWRKY proteins, we used AtWRKY domains as query sequences to constructed phylogenetic tree. MAFFT 7.0 program was applied to multiple sequences alignment (Katoh and Standley, 2013). The phylogenetic trees were inferred using MEGA 6.0 with the neighbor-joining method (Tamura et al., 2013). Bootstrap values were calculated for 1000 iterations.

All *GmWRKY* genes were mapped to soybean chromosomes based on information available from SoyBase (http://soybase.org/). The map was drafted using MapInspect software (http://mapinspect.software.informer.com/).

Soybean EST sequences were downloaded from GenBank (http://www.ncbi.nlm.nih.gov/est). *GmWRKY* genes were used as query to blast against soybean ESTs using BLASTN with sequence similarity >96%, length >= 200 bp, and *E*-value <10^−10^.

### Analysis of codon usage bias

Codon usage bias was derived from cDNA sequences encoding full-length proteins. To avoid sampling bias, CDS sequences were filtered based on the following criteria: (1) full-length CDS sequences shorter than 300 bp were excluded from this analysis; (2) the presence of a start codon (ATG) beginning and a stop codon (TAA, TAG, and TGA) ending in each CDS was required. Codon usage bias was calculated from sequences using the Codon W 1.4 program (http://codonw.sourceforge.net) and Perl scripts.

### Expression profiles of *GmWRKY* genes

The normalized data (Reads/kb/Million, RPKM) for six different tissues from different growth periods was reported by Severin et al. (2010), and available from SoyBase website. A gene was considered expressed if the RPKM value was greater than or equal to two in an expression atlas (Belamkar et al., 2014). The RPKM normalized read count data of expressed genes was log_2_-transformed and displayed in the form of heatmaps in R script. To survey the involvement of *GmWRKY* genes in dehydration and salt stress responses, transcriptome sequencing data of soybean under dehydration, and salt stress at three time points (1, 6, and 12 h) was downloaded from described by Belamkar et al. (2014). The satisfied criteria of differentially expressed genes was as follows: (1) *P*-value adjusted for multiple testing correction using Benjamini and Hochberg method (Benjamini and Hochberg, 1995) to be <0.05, (2) two fold or greater fold change, (3) residual variance quotients of both the control and treatment samples of <20.

### *cis*-Acting elements analysis in *GmWRKY* promoters

PlantCARE online program (http://bioinformatics.psb.ugent.be/webtools/plantcare/html/) was used to predict *cis*-acting elements in *GmWRKY* promoters. Sequence of 2000 bp upstream of the start codon was used for *cis*-acting elements analysis. The sequences were obtained from Phytozome 10.0 database.

### Plant material and stress treatments

The seeds of soybean (Williams 82) were germinated on wet filter paper in growth chamber at 28°C, and grown for 2 weeks at room temperature (about 32°C). For salt treatment, roots of the seedlings were transferred into 100 mM NaCl solution at room temperature. For dehydration treatment, seedlings were removed from the wet filter paper and kept in air at room temperature. Roots were harvested after 0, 6, 12, 24, and 48 h exposure to NaCl and dehydration treatment.

### Quantitative real-time RT-PCR

Total RNA was extracted using the CTAB method (Chang et al., 1993). The first-strand cDNAs were synthesized using 2 μg RNA using the Reverse Transcriptase M-MLV System (Takara, Dalian, China). Primers for quantitative real-time RT-PCR (qRT-PCR) analysis were listed in Table S1. Metalloprotease gene (forward primer: 5′-ATGAATGACGGTTCCCATGTA-3′; reverse peimer: 5′-GGCATTAAGGCAGCTCACTCT-3′) was used as a reference gene (Libault et al., 2008). qRT-PCR was carried out using Fast Start Universal SYBR Green Master (ROX) with a 7500 real-time PCR machine (ABI). The reactions were carried out using the following program: 95°C for 30 s, followed by 40 cycles of 95°C for 5 s, 60°C for 30 s. Melting curve was generated from the following program: 95°C for 15 s, 60°C for 60 s, 95°C for 30 s, and 60°C for 15 s. Three biological replicates were used for qRT-PCR analysis. The ^ΔΔ^C_t_ method was used for quantification (Livak and Schmittgen, 2001).

## Results

### WRKY proteins in soybean

We predicted 192 WRKY sequences from soybean genome using HMMER program. Seven of these protein sequences were not WRKY sequences, and other nine proteins were excluded in this study because each of them contained an incomplete WRKY domain (Table S2). The remaining 176 proteins were identified as putative WRKY proteins in soybean genome Wm82.a2v1. GmWRKY proteins could be divided into three groups based on the number of WRKY domains and the type of zinc-finger structure. Group I, II, and III contained 32, 120, and 24 proteins, respectively (Table 1 and Table S2). The conserved WRKY domain from *Arabidopsis* and soybean were used to reconstruct a neighbor-joining phylogenetic tree (Figure S1). Soybean Group II WRKY proteins could be further divided into five subgroups, namely IIa, IIb, IIc, IId, and IIe, and each containing 12, 30, 41, 15, and 22 sequences, respectively (Figure S1 and Table 1). Notably, we identified one novel WRKY (Glyma.14G085500), which was named GmWRKY183 (Table S2).

**Table 1:** **Number of ***WRKY*** genes identified in plants**.

**Species**	**Group I**	**Subgroup IIa**	**Subgroup IIb**	**Subgroup IIc**	**Subgroup IId**	**Subgroup IIe**	**Group III**	**Total**	**Genome size(Mb)**
*Glycine max*	32	12	30	41	15	22	24	176	978
*Arabidopsis thaliana*^a^	13	4	7	18	7	9	14	72	125
*Brachypodium distachyon*^b^	17	3	6	21	6	10	23	86	272
*Cucumis sativus*^a^	10	4	4	16	8	7	6	55	367
*Lotus japonicus*^c^	12	5	8	13	5	9	7	59	472
*Medicago truncatula*^c^	19	6	8	19	8	8	13	81	375
*Oryza sativa*^a^	34	4	8	7	11	0	36	100	480
*Populus trichocarpa*^d^	50	5	9	13	13	4	10	104	485
*Vitis vinifera*^a^	12	4	8	16	7	6	6	59	487
*Gossypium raimondii*^e^	20	7	16	26	16	13	14	112	737
*Gossypium arboreum*^e^	19	7	16	26	14	13	14	109	1746

a Data from Ling et al. (2011)

b data from Tripathi et al. (2012)

c data from Song et al. (2014)

d data from He et al. (2012)

e *data from Ding et al. (2015)*.

The WRKYGQK sequence is considered to be important for recognizing and binding to W-box elements (C/TTGACT/C) in the promoter of target genes (Eulgem et al., 2000). Previous studies showed that variation of WRKYGQK sequences was observed in many species (Wu et al., 2005; He et al., 2012; Liu et al., 2014; Song et al., 2014). Besides the most common WRKYGQK sequence, we found seven other heptapeptide variants in GmWRKY, namely, WRKYGKK, WRKYGEK, WRKYGKR, WRKYEDK, WKKYGQK, WRKYGKK, and WHQYGLK (Table S2). WRKYGKK sequence appeared with the highest frequency among them, which belong to subgroup IIc. WRKYGKK sequence is the most common variant not only in soybean, also in *Solanum lycopersicum* (Huang et al., 2012), *L. japonicus* (Song et al., 2014), and *Brassica oleracea* var. *capitata* (Yao et al., 2015). WRKYGKK sequence in tobacco WRKY could bind specifically to WK-box (TTTTCCAC), which was significantly different from the consensus sequence of W-box (van Verk et al., 2008). Three WRKYGEK sequences were found in GmWRKY5 (group I), GmWRKY67 (group I), and GmWRKY25 (subgroup IIc), respectively. Two WKKYGQK sequences were identified in GmWRKY80 (subgroup IIa) and GmWRKY102 (subgroup IIa), respectively. WRKYGKP, WRKYEDK, and WHQYGLK were identified in GmWRKY91 (group III), GmWRKY148 (group I), and GmWRKY130 (subgroup IIc), respectively (Table S2). WHQYGLK sequence, with the most divergent variation among these seven variants, might execute new biological functions.

**Figure 1:**
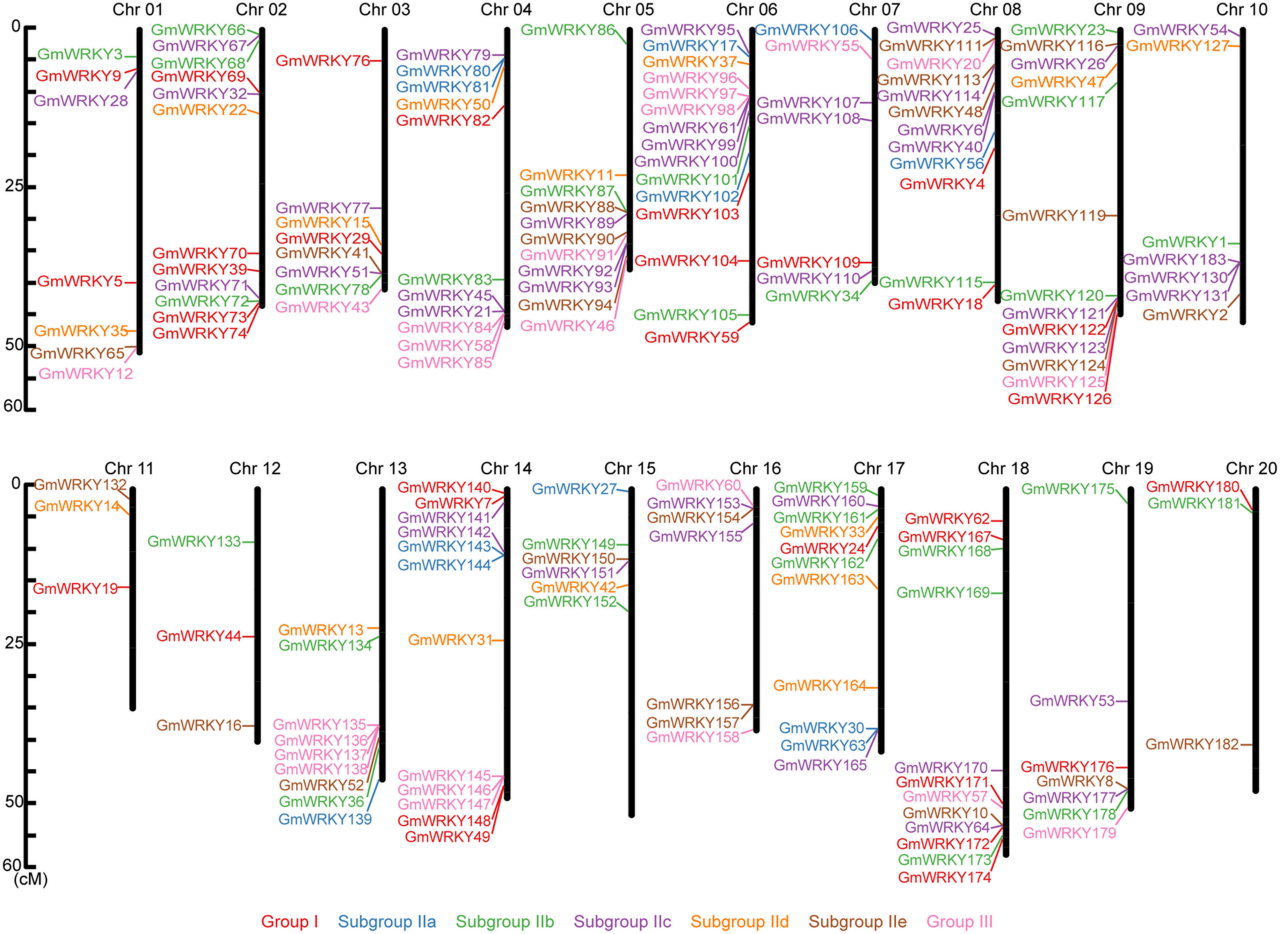
**Chromosomal location of ***GmWRKY*** genes**. The chromosome numbers were shown at the top of each chromosome (black bars). The names on the left side of each chromosome correspond to the approximate location of each *WRKY* gene.

These 176 *GmWRKY* genes were randomly distributed throughout the 20 soybean chromosomes (Figure 1). There were more *GmWRKY* genes (15 genes) on chromosome 6, and chromosome 11, 12, and 20 each contained only three *GmWRKY* genes. We found that more *GmWRKY* genes were located at both ends of chromosomes (Figure 1). No group I *GmWRKY* gene was found in chromosome 5, 10, 13, 15, and 16. Group III *GmWRKY* gene was not detected in chromosome 2, 10, 11, 12, 15, 17, and 20. Group II genes were distributed in 20 chromosomes. Chromosome 10 and 15 contained only group II genes.

Publicly available ESTs were considered as a useful source for gene expression study (Ohlrogge and Benning, 2000). A total of 1,468,526 soybean ESTs were downloaded from GenBank. A total of 127 *GmWRKY* genes were obtained from these ESTs which were generated from leaf, seed, and other tissues (Table S3). These ESTs were sequenced from soybean plants under different stresses. We found that the expression of *GmWRKY4, 5, 6, 9, 31, 46, 50, 56, 96, 106, 155*, and *160* genes was responsive to water deficit stress, and *GmWRKY10, 49, 121*, and *155* genes showed altered expression in response to salt stress.

### Codon usage bias analysis

**Figure 2:**
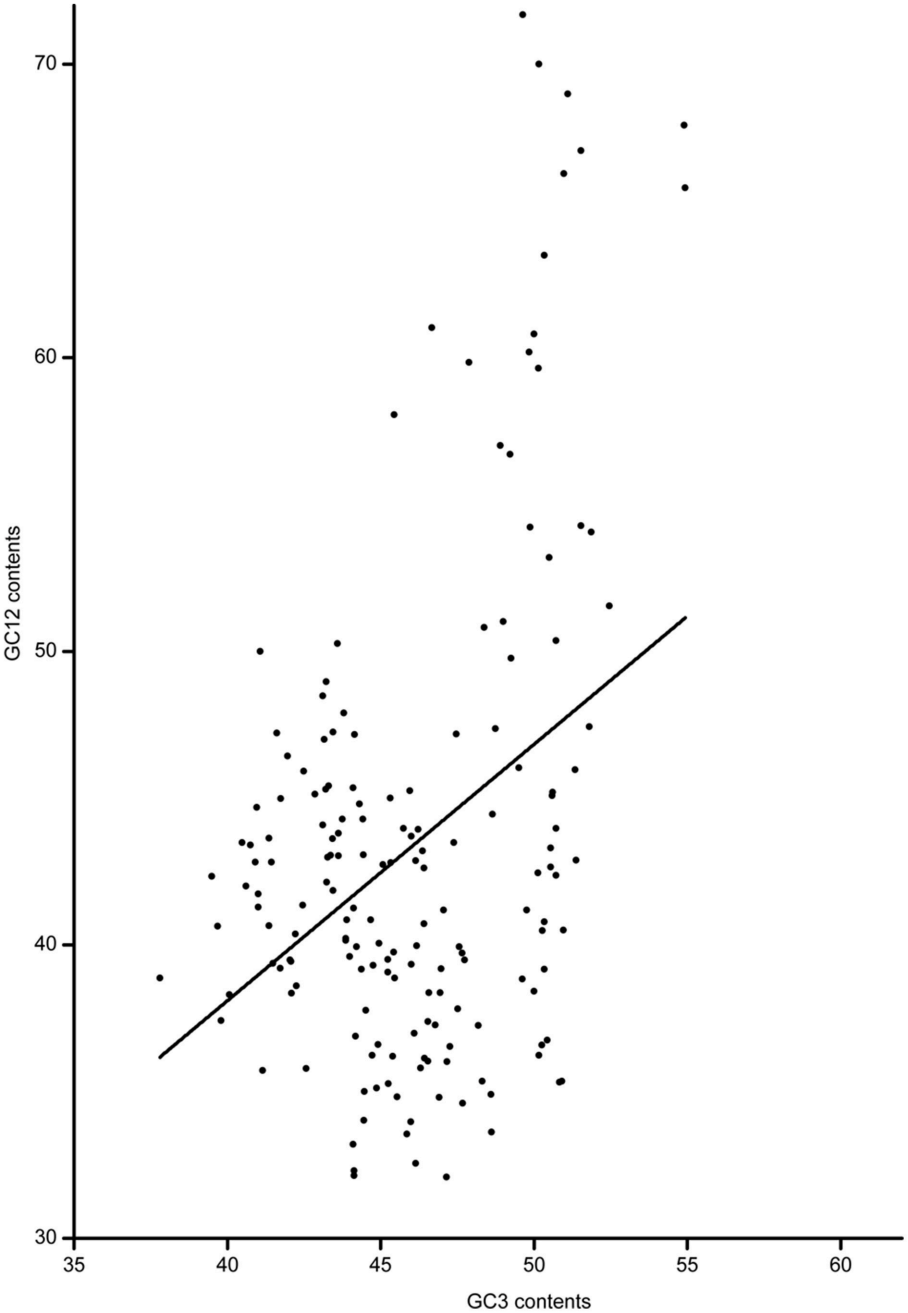
**Neutrality plots (GC12 vs. GC3s)**. The regression line: y = 0.87345X + 3.15405.

**Figure 3:**
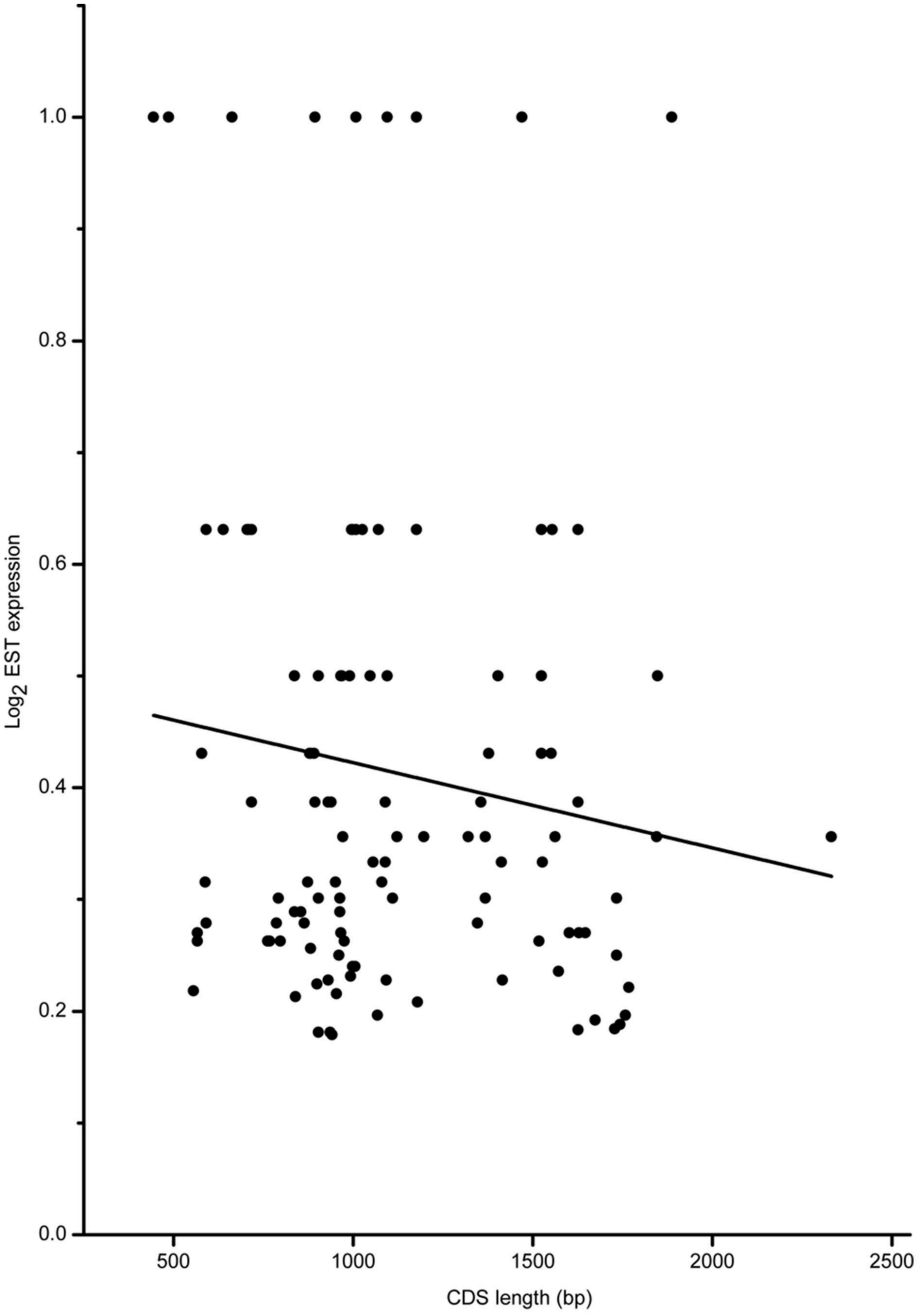
**Relationship between gene expression and CDS length**. The regression line: y = −0.000000762534*X* + 0.49866.

**Figure 4:**
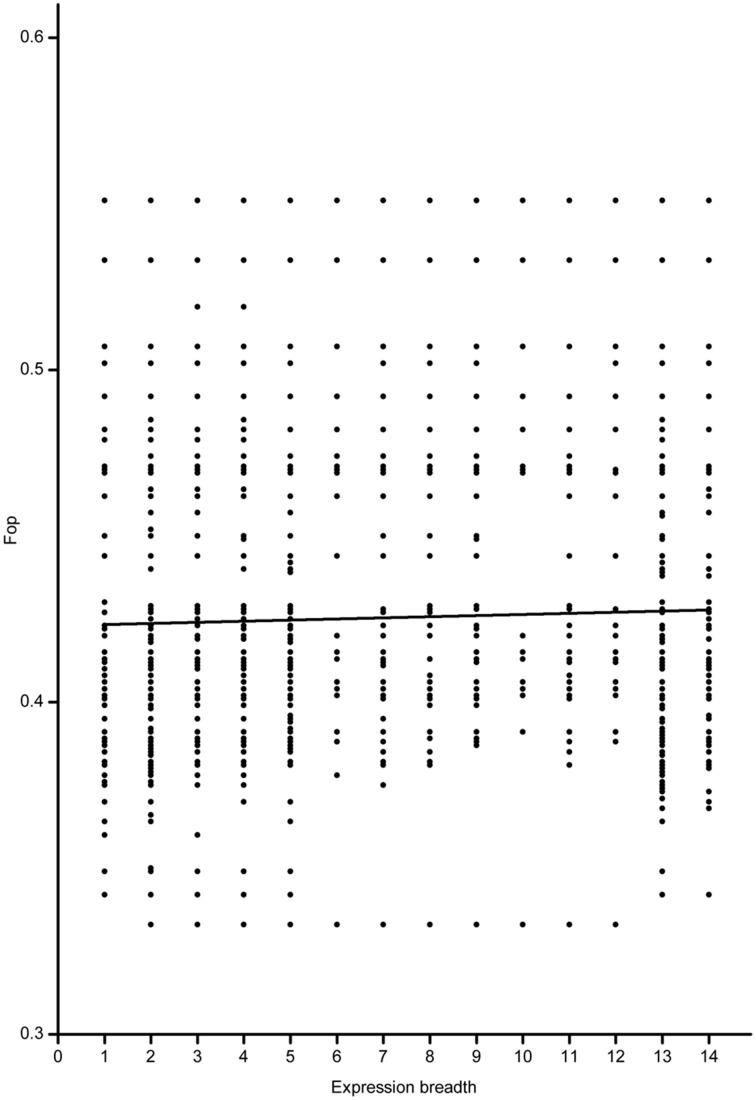
**Relationship between fop and expression breadth**. The regression line: y = 0.000337875*X* + 0.42302. The number of 1–14 indicated yongg_leaf, flower, one.cm.pod, pod. shell. 10 DAF, pod. shell. 14 DAF, seed. 10 DAF, seed. 14 DAF, seed. 21 DAF, seed. 25 DAF, seed. 28 DAF, seed. 35 DAF, seed. 42 DAF, root, and nodule, respectively.

Based on the full-length CDS sequences of 171 *GmWRKY* genes, GC content in three codon positions was analyzed using the Perl scripts. The GC1 value (48.30) was higher than that of GC2 (43.80) and GC3 (43.37). The average GC content of all codons was 45.15. The AT content (54.85) was higher than GC content in *GmWRKY* genes. **N**eutrality plots (GC12 vs. GC3) were used to analyze the relationship among three codon positions. We detected a positive correlation (*P* < 0.05) between GC12 and GC3 (Figure 2), indicating GC mutational bias leading to similar GC content in all codon positions. Optimal codons of *GmWRKY* genes showed a greater preference for a C or G in the third base position (Table S6), while accounting for its lower GC in third base when compared with the AT content. Song et al. (2015) reported that AT content were higher than GC content in *MtWRKY* genes, but the 3rd position exclusively used G or C in optimal codons. We found a significant negative correlation (*P* < 0.05) between EST expression data and length of CDS sequences (Figure 3), indicating a tendency of higher level expression for genes with shorter CDS, and lower level expression for longer CDS genes. The correlation between codon bias and expression breadth was significant positive (*P* < 0.05; Figure 4), indicating that *GmWRKY* with larger expression breadth showed a high degree of codon usage bias.

### RNA-seq analysis of *GmWRKY* expression

**Figure 5:**
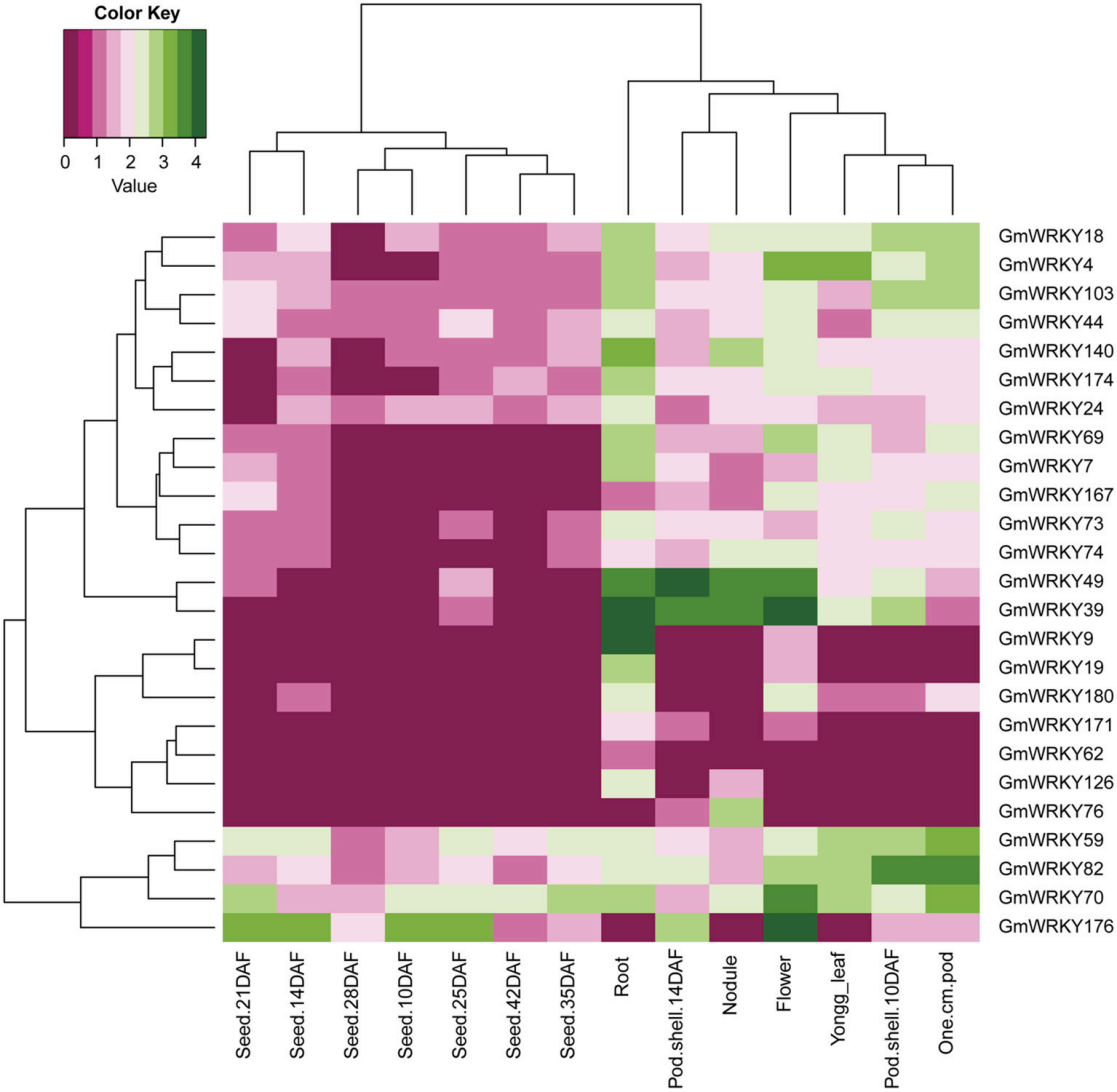
**Expression pattern of ***GmWRKY*** group I genes in different tissues**. The Reads/Kb/Million (RPKM) normalized values of expressed genes was log_2_-transformed. The abbreviation “DAF” in the tissue label indicates “Days after flowering.”

**Figure 6:**
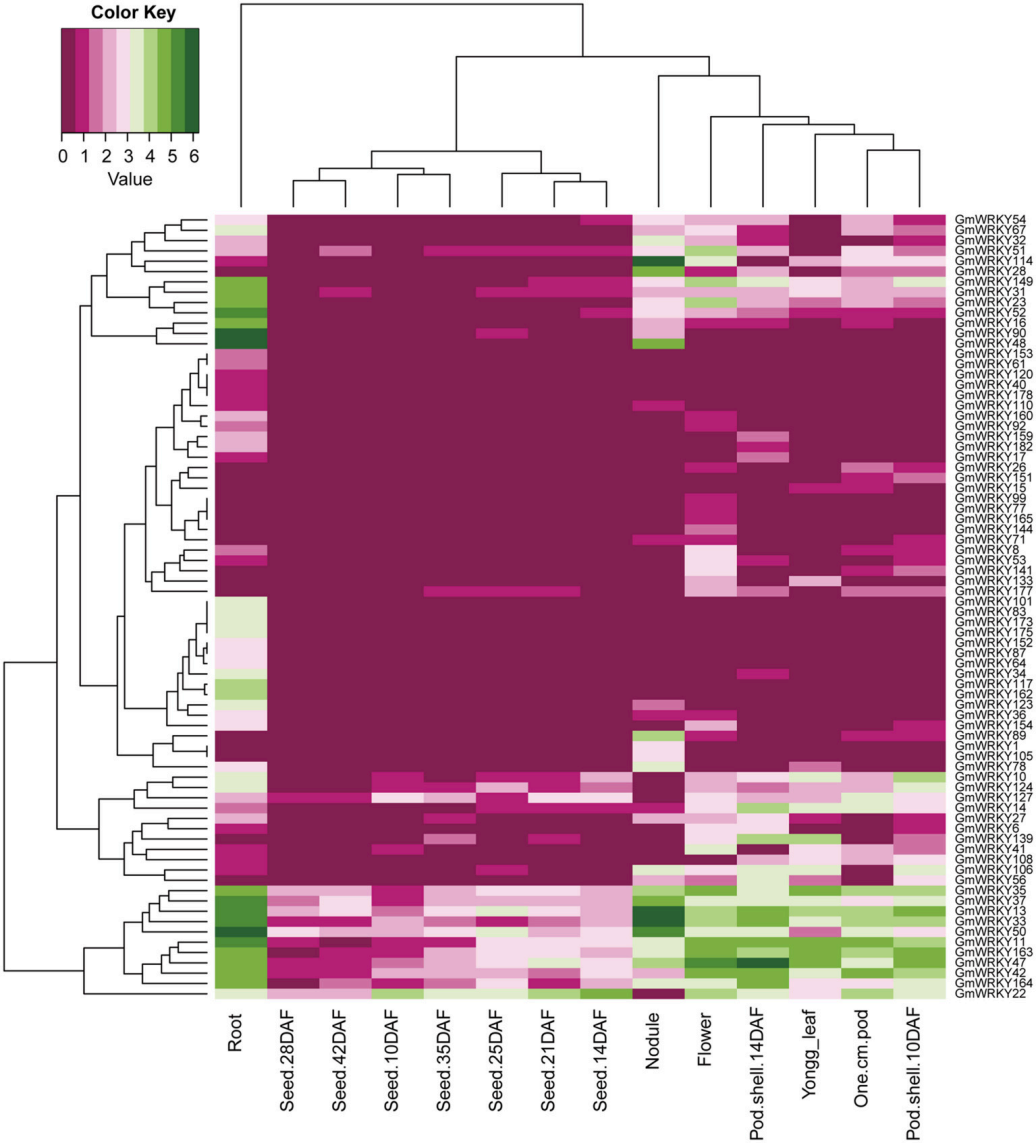
**Expression pattern of ***GmWRKY*** group II genes in different tissues**. The Reads/Kb/Million (RPKM) normalized values of expressed genes was log_2_-transformed. The abbreviation “DAF” in the tissue label indicates “Days after flowering.”

**Figure 7:**
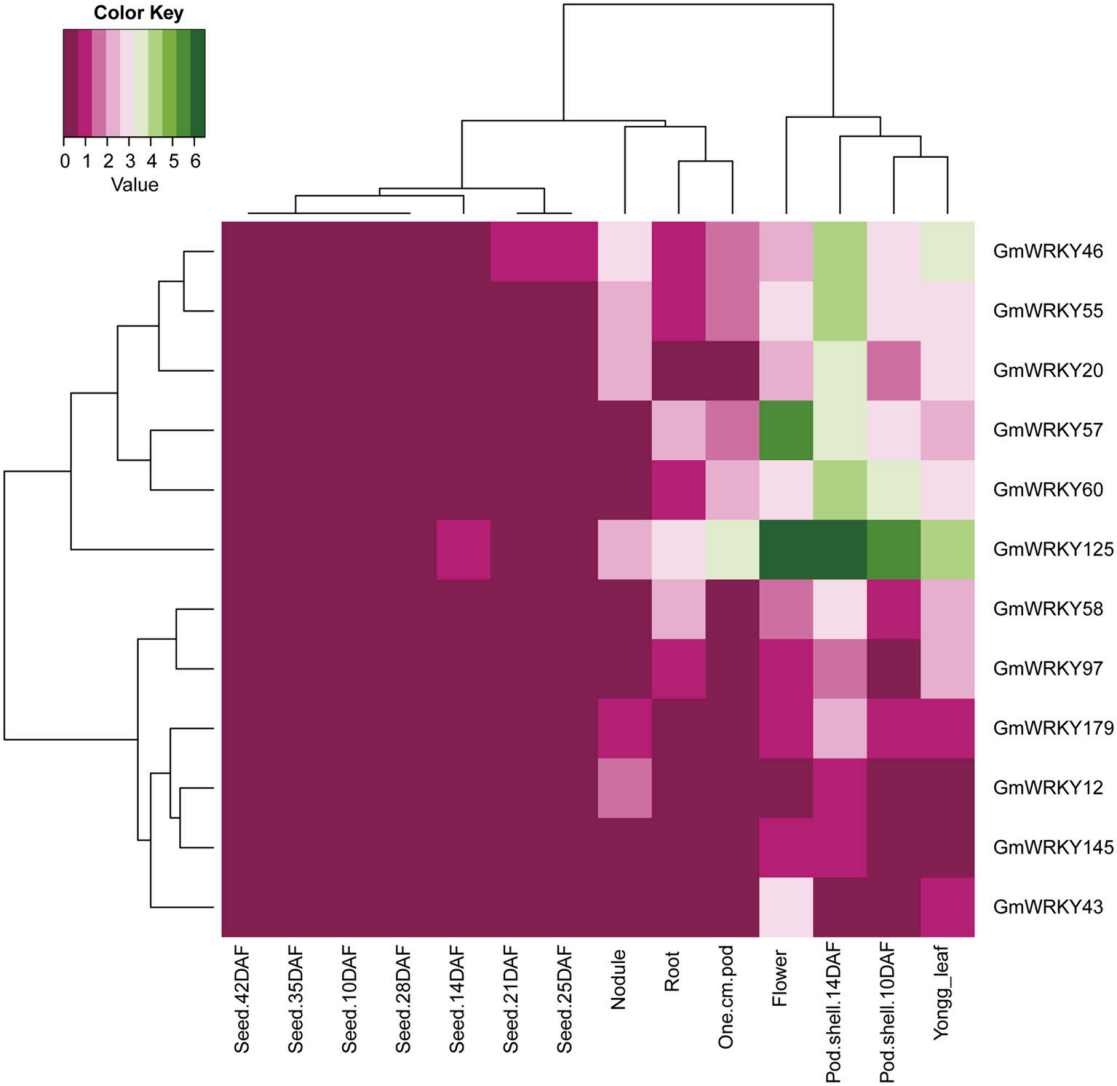
**Expression pattern of ***GmWRKY*** group III genes in different tissues**. The Reads/Kb/Million (RPKM) normalized values of expressed genes was log_2_-transformed. The abbreviation “DAF” in the tissue label indicates “Days after flowering.”

One hundred and three *GmWRKY* genes were expressed at various developmental stages in leaf, flower, pod, seed, root, and nodule of soybean. Fifteen, 76, and 12 of *GmWRKY* genes belonged to group I, II, and III, respectively. Group I genes were highly expressed in root, leaf, flower, nodule, and pod. Five group I genes (*GmWRKY44, 59, 70, 82*, and *103*) expressed in all these tissues at various development stages (Figure 5). Group II *GmWRKY23, 31, 52*, and *149* genes were mainly expressed in leaf, flower, nodule, and pod. *GmWRKY11, 13, 33, 35, 37, 42, 47, 50*, and *127* genes in group II were expressed in all six tissues at various development stages (Figure 6). Group III *GmWRKY* genes were mainly expressed in root, leaf, flower, nodule, and pod, for example, *GmWRKY46, 55*, and *125* genes. However, for an individual group III gene, none of them was detected in all six tissues (Figure 7 and Table S2). Experimental evidence is lacking for the involvement of *WRKY* gene in floral development or organogenesis. However, most *GmWRKY* genes were highly expressed in flower, suggesting their roles in floral development.

**Figure 8:**
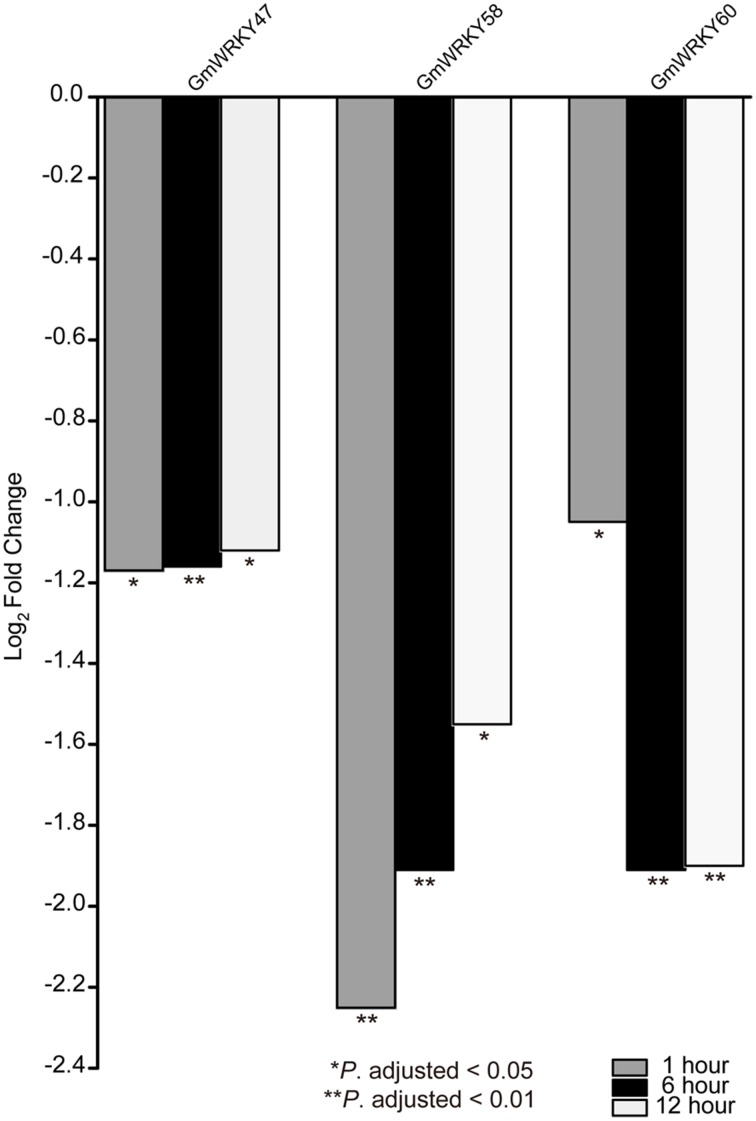
**Different expression of ***GmWRKY*** genes in three time point under dehydration stress based on RNA-seq data**.

**Figure 9:**
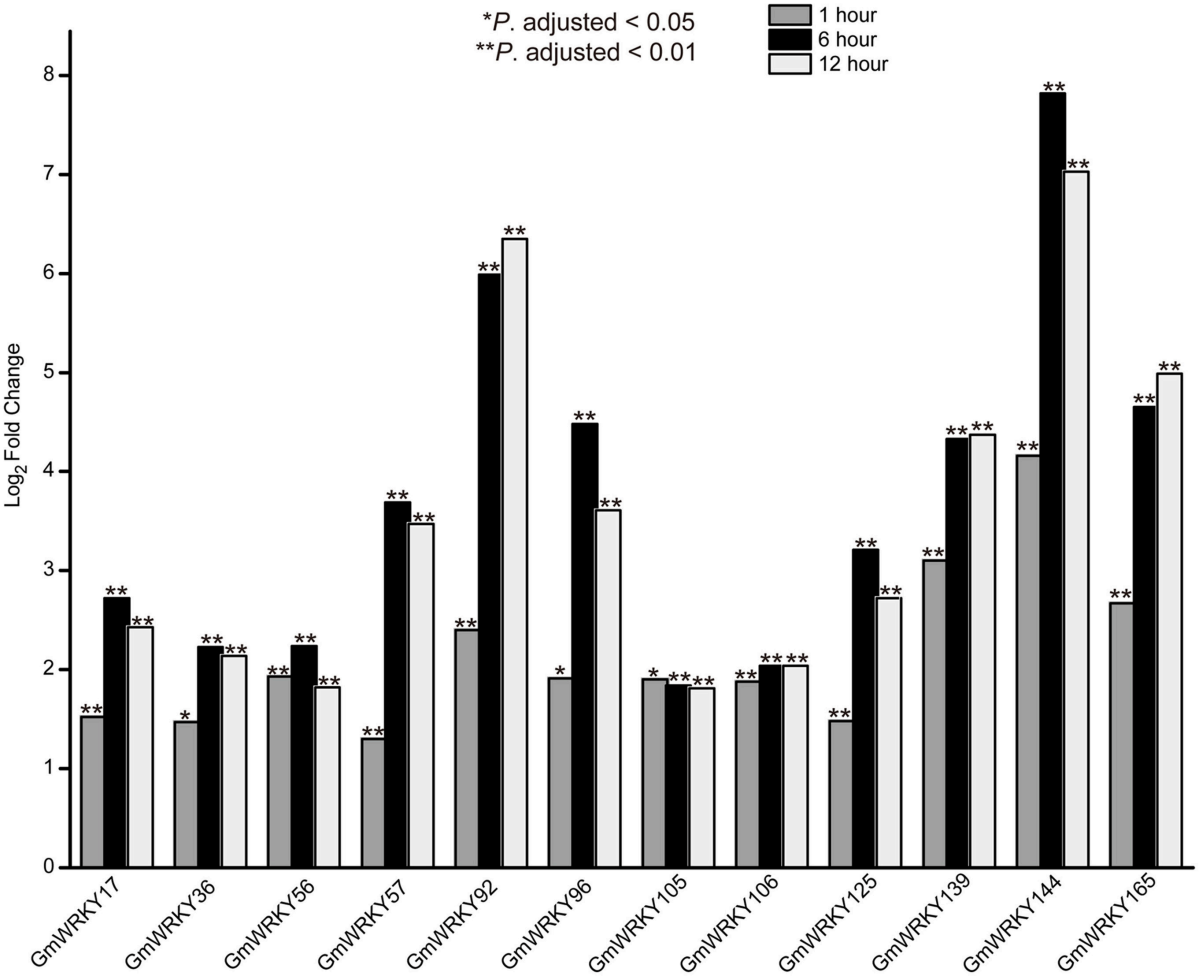
**Different expression of ***GmWRKY*** genes in three time point under salt stress based on RNA-seq data**.

RNA-seq data was used to screen *GmWRKY* genes that are responsive to dehydration and salt stress. A total number of 31 and 65 *GmWRKY* genes are considered differentially expressed at least at one of the three time points under dehydration or NaCl treatment, respectively (Tables S4, S5). Dehydration induced down-regulation of most *GmWRKY* genes except *GmWRKY56, 106, 120*, and *139* genes at 1 h (Table S4). *GmWRKY47, GmWRKY58*, and *GmWRKY60* genes were differentially expressed at least at one of three time points under dehydration. These genes were significantly down-regulated (Figure 8). NaCl treatment resulted in up-regulation of most *GmWRKY* genes (Figure 9 and Table S5). Twelve *GmWRKY* genes were differentially expressed at all three time points under salt (Figure 9). Most of the differentially expressed genes belong to group II (dehydration: 17/31; salt: 45/65), and followed by group III (dehydration: 9/31; salt: 13/65). Less group I genes (dehydration: 5/31; salt: 7/65) were found responsive to salt and dehydration stresses. These results were consistent with results from cotton *WRKY* that most group II and III *GhWRKY* genes are highly expressed under stress condition (Dou et al., 2014).

### *cis*-Acting element analysis

Ninety-five *GmWRKY* genes are differentially expressed under dehydration and/or salt stresses (Tables S4, S5). Removal of three genes (*GmWRKY175, GmWRKY53*, and *155*) with incomplete sequences or low quality sequences, we extracted 2000 bp promoter regions of 92 *GmWRKY* genes. Various types of *cis*-acting elements were detected in the promoter region of 92 *WRKY* genes, suggesting that the same type of *GmWRKY* could perform different functions. Most of W-box elements could be distributed in promoters of *GmWRKY* (Tables S7, S8). Some *WRKY* genes, contained majority of W-box elements, are auto-regulated by itself and cross-regulated, indicating a self-feedback or mutual manipulation channel might exist among *WRKY* genes (Chi et al., 2013). *AtWRKY18, 40*, and *60* genes were reported to be self- and cross-regulated based on W-box elements (Yan et al., 2013). Similarly, *OsWRKY24, 53*, and *70* genes were predicted self- and cross-regulated according to the presence of W-box clusters in their promoters (Zhang et al., 2015). Based on these reports, we speculated that the presence of W-box elements in *GmWRKY* gene might have the similar regulatory mode. ABRE and MBS elements that respond to dehydration or salt stress were distributed in promoter region of most *GmWRKY* genes (Tables S7,S8). These results suggested that *GmWRKY* genes were transcriptionally regulated upon dehydration and salt stress.

### *GmWRKY* genes expression in response to dehydration and salt stress using qRT-PCR

**Figure 10:**
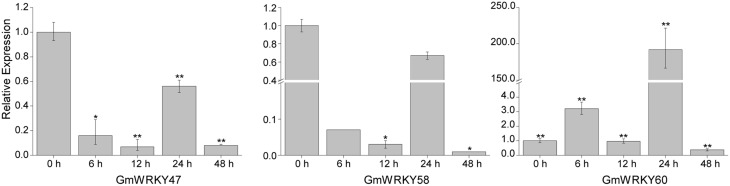
**Expression pattern of selected ***GmWRKY*** genes under dehydration stress**. The Y-axis indicates the relative expression; X-axis (0, 6, 12, 24, and 48 h) indicates hours of dehydration treatment. The standard errors are plotted using vertical lines. ^*^significant difference at *P* < 0.05, ^**^significant difference (*P* < 0.01).

**Figure 11:**
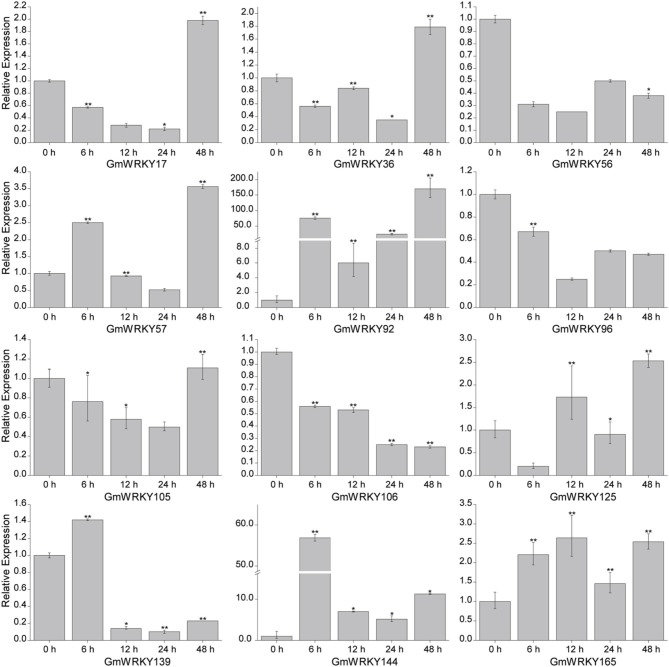
**Expression pattern of selected ***GmWRKY*** genes under salt stress**. The Y-axis indicates the relative expression; X-axis (0, 6, 12, 24, and 48 h) indicates hours of salt treatment. The standard errors are plotted using vertical lines. ^*^significant difference at *P* < 0.05, ^**^significant difference (*P* < 0.01).

To validate the expression patterns of *GmWRKY* genes revealed by RNA-seq, 15 *GmWRKY* genes were selected for expression analysis by qRT-PCR. The RNA-seq results showed that 3 and 12 *GmWRKY* genes were responsive to dehydration and salt stress at all three time points, respectively. The qRT-PCR results showed the expression of *GmWRKY47* and *58* genes was down-regulated at 6, 12, 24, and 48 h under dehydration stress (Figure 10). Expression of *GmWRKY60* gene was significantly increased at 6 and 24 h, but significantly suppressed at 48 h under dehydration stress. The results showed that *GmWRKY47* and *58* genes were consistent with RNA-seq data while *GmWRKY60* gene was not. The expression of *GmWRKY92, 144*, and *165* genes was enhanced at all time points using qRT-PCR (Figure 11). The results were consistent with the RNA-seq data. Unexpectedly, the expression of remaining nine *GmWRKY* genes was not consistent with RNA-seq data. For example, the expression of *GmWRKY56, 96*, and *106* genes was reduced at all four time points (Figure 11), but they showed up-regulation in RNA-seq analysis.

## Discussion

### Identification and characterization of WRKY

In this study, we identified 176 WRKY proteins in soybean Wm82.a2v1 genome. Compared to present study, Zhou et al. (2008) found 64 GmWRKY sequences and Bencke-Malato et al. (2014) identified 149 GmWRKY in Wm82.a1v1 genome. The new assembled Wm82.a2v1 genome constructed using the latest ARACHNE assemble is more accurate. Annotation of eight GmWRKY has been changed in the new assembled genome (Table 2). For example, GmWRKY148, 171, and 172 belonged to subgroup IIc in Wm82.a1v1, but belong to group I in Wm82.a2v1. Moreover, GmWRKY57 and 67 were classified into group I in Wm82.a1v1, but they were in group III and subgroup IIc in Wm82.a2v1, respectively. These results showed that there were differences between Wm82.a1v1 and Wm82.a2v1 genomes. Therefore, it is important to update the global information of GmWRKY in the latest version of soybean genome.

**Table 2:** **Annotation revised in ***GmWRKY*** genes**.

**Name**	**Previous annotation**	**Present annotation**
GmWRKY10	III	IIe
GmWRKY57	I	III
GmWRKY64	IIe	IIc
GmWRKY67	I	IIc
GmWRKY148	IIc	I
GmWRKY170	IIb	IIc
GmWRKY171	IIc	I
GmWRKY172	IIc	I

Compared to other plants, soybean genome contained the highest number of WRKY members. The expansion of WRKY proteins in soybean might be due to the following reasons. (1) soybean genome experienced at least three rounds of whole genome duplication (WGD) events that could produce a large number of paralogs (Conant and Wolfe, 2008). The dicotyledons, such as *Arabidopsis*, grape, soybean, and *M. truncatula*, share a general “gamma” genome triplication event about 117 million years ago (Mya; Schmutz et al., 2010). Subsequently, soybean and *Medicago* experience a common legume-specific WGD event around 59 Mya, and soybean has undergone an additional glycine-specific genome duplication event approximately 13 Mya (Schmutz et al., 2010). (2) The number of duplicated genes was mainly determined by segmental duplication events because genes generated by segmental duplicated events have more chance to be retained (Wang et al., 2005). Tandem duplication events play an important role in generating new duplicated genes (Cannon et al., 2004), whereas segmental duplication events may widely distribute duplicated genes across the genome (Baumgarten et al., 2003). Segmental duplication events could result in lost of many functional redundant genes to avoid fitness cost (Song et al., 2014). Yin et al. (2013) found that *GmWRKY* genes were generated mainly through segmental duplication events, which may lead to neofunctionalization or subfunctionalization (Moore and Purugganan, 2005). Gene duplication events could improve plant resistance to pathogens by allowing the functional diversification of genes (Moore and Purugganan, 2005). It was reported that 75 *GmWRKY* genes were involved in response to fungal infection (Bencke-Malato et al., 2014). (3) Positive selection play a key role in preserving duplicated genes, and can act at very early stage of gene duplication process (Moore and Purugganan, 2005). Site model and branch-site model analysis showed that group I, IIc, IIe, and III GmWRKY underwent positive selection (Yin et al., 2013). Positive selection promotes constant expansion of GmWRKY. Similarly, group IIc and III WRKY from eggplant and turkey berry were speculated to undergo positive selection (Yang et al., 2015). In contrast, Group III WRKY from *L. japonicus* (Song et al., 2014), *M. truncatula* (Song and Nan, 2014), and *Cucumis sativus* (Ling et al., 2011) appear to be under purifying selection. Purifying selection may generate genes with conserved functions or pseudogenization (Zhang, 2003). The genome size and the number of WRKY family members are not necessarily correlated. For example, the genome size of soybean is 978 Mb containing 176 WRKY, while the genome size of *Gossypium arboretum* is 1746 Mb containing 109 WRKY. The genome size of *G. arboreum* (1746 Mb) was about three times greater than the *Populus trichocarpa* genome size (485 Mb). These two plants have approximately same number of WRKY (109 vs. 104; Table 1).

### *GmWRKY* expression in different tissues and stress conditions

Dou et al. (2014) reported that most *Gossypium hirsutum WRKY* genes expressed at low levels in all developmental stages, while a few *GhWRKY* expressed highly in specific organs. Huang et al. (2012) found that 10 *S. lycopersicum WRKY* genes were constitutively expressed in nearly all tissues. Our results showed that *GmWRKY* genes expressed with distinct temporal and spatial patterns. Sixteen *GmWRKY* genes from group II were expressed in root, flower, or nodule with tissue-specific manner (Figure 6). The expression of a particular *GmWRKY* gene in a given tissue may differ at different developmental stages. For example, the expression of *GmWRKY71* and *154* genes was observed in 10 DAF pod shell, but hardly detected in other developmental stages. *GmWRKY54, 62, 125*, and *180* genes were expressed in 14 DAF seeds, but not in other seed developmental stages (Figure 6).

Although a little evidence demonstrated the involvement of *GmWRKY* genes in flower development, many *GmWRKY* genes were highly expressed in flowers (Figures 5–7). Recently, Luo et al. (2013b) reported that heterologous expression of *WRKY20* from *Glycine soja* in *Arabidopsis* resulted in earlier flower. *GsWRKY20* is orthologous of *AtWRKY53, GmWRKY20, 46*, and *55*. We found that *GmWRKY20, 46*, and *55* genes were highly expressed in flower (Figure 7). This was consistent with the results from *Brassica rapa*, where most of *BrWRKY* highly expressed in flower buds (Kayum et al., 2015). Here, we speculated that these three *GmWRKY* genes might play roles in flower development.

Stomata and roots were involved in plant responses to dehydration and salt stress (Song et al., 2009; Chen et al., 2012; Belamkar et al., 2014). The RNA-seq data showed that 15 *GmWRKY* genes were differentially expressed under dehydration and salt stress in root. Zhou et al. (2008) found that *GmWRKY54* genes confer tolerance to salt and drought stresses in *Arabidopsis*, possibly through the regulation of *DREB2A* and *STZ/Zat10* genes. Heterologous expression of *GmWRKY13* genes could increase the sensitivity of *Arabidopsis* to salt tolerance (Zhou et al., 2008). However, we failed to detect the response of these two genes to dehydration and salt stress. The possible explanation might be due to tissue-specific regulation; *GmWRKY13* and *54* genes were cloned from leaf, while the RNA-seq data were from root.

Under dehydration stress, *GmWRKY47, 58*, and *60* genes were considered as differentially expressed genes. Their orthologous genes in *Arabidopsis* are *AtWRKY11, 41*, and *70*, respectively (Table S2). *WRKY11* from *Vitis vinifera* is orthologous gene of *AtWRKY11*. Transgenic *Arabidopsis* expressing *VvWRKY11* showed higher tolerance to drought stress, indicating its involvement in response to dehydration stress (Liu et al., 2011). Overexpression of *GsWRKY20* (orthologous of *AtWRKY 70*) in *Arabidopsis* and *Medicago sativa* could increase drought tolerance of the transgenic *Arabidopsis*, and enhance salt and drought tolerance of transgenic *Medicago* (Luo et al., 2013a; Tang et al., 2014). *VvWRKY11* and *GsWRKY20* promoted dehydration tolerance. However, our results from qRT-PCR and RNA-seq data showed that *GmWRKY47* (*AtWRKY11*) and *60* (*AtWRKY70*) genes were negatively regulated by dehydration. *AtWRKY41* (orthologous of *GmWRKY58*) could promote disease resistance (Higashi et al., 2008). It could promote seed dormancy through regulation of *ABI3* gene (Ding et al., 2014). We first reported the observation that *GmWRKY58* gene was involved in dehydration response.

Twelve *GmWRKY* genes were differentially expressed under salt stress. Their orthologous genes are *AtWRKY6, 30, 40, 50, 51*, and *70* in *Arabidopsis*, respectively (Table S2). The orthologous genes of *AtWRKY40* are *GmWRKY17, 56, 106, 139*, and *144*; Both of *GmWRKY36* and *105*, which are orthologous genes of *AtWRKY6*; and *GmWRKY57* and *125* shared a common orthologous gene, *AtWRKY70*. *AtWRKY6* was identified as target gene of *AtWRKY53* (Miao et al., 2004). The expression of *AtWRKY53* was up-regulated in *Arabidopsis sos2* mutant under salt stress (Kamei et al., 2005), indicating its involvement in salt tolerance. Scarpeci et al. (2013) showed that overexpression of *AtWRKY30* enhanced salt tolerance in *Arabidopsis* during early growth stages. ChIP experiments showed that *AtWRKY40* directly targeted a number of known ABA-responsive genes, including *ABI4, ABI5, ABF4, MYB2, DREB1A*, and *RAB18* genes (Shang et al., 2010), indicating that *AtWRKY40* could promote salt tolerance. Previous study showed that *AtWRKY50* and *51* played crucial roles in jasmonic acid (JA) pathway (Chen et al., 2012), which was a key component in pathogen tolerance. We found the expression of *GmWRKY92* (orthologous of *AtWRKY51*) and *165* (orthologous of *AtWRKY50*) was up-regulated under 100 mM NaCl treatment (Figure 11), indicating their involvement in multiple stress responses.

There are many opposite results between qRT-PCR and RNA-seq analysis. We speculated that growing condition and seed dormancy time probably caused different expression pattern between qRT-PCR and RNA-seq data. *WRKY* genes are involved in varies of plant development, including seed dormancy and germination (Zhang et al., 2004; Zentella et al., 2007; Zou et al., 2008). Moreover, some *WRKY* genes have different functions (Rushton et al., 2010; Tang et al., 2013), indicating the selected *GmWRKY* genes probably involved in multiple biological pathway.

## Conclusions

In this study, we identified 176 GmWRKY proteins in soybean genome (Wm82.a2v1) using bioinformatics approach. There are more WRKY proteins in soybean genome than other plant species. We found that no positive correlation exists between the genome size and the number of WRKY. Expression analysis showed that some *GmWRKY* genes were involved in response to dehydration and salt stress. Our results will be helpful for understanding the roles of *WRKY* gene family in soybean.

## Author contributions

HS wrote the manuscript and performed the field and laboratory assays. PW performed the phylogentic analysis and reviewed the manuscript. LH, SZ, CZ, HX, and PL performed the RNA-seq analysis. YZ and XB provied help in analysis of qRT-PCR. XW served as the principal investigator, facilitated the project, and assisted in manuscript preparation and revision.

### Conflict of interest statement

The authors declare that the research was conducted in the absence of any commercial or financial relationships that could be construed as a potential conflict of interest.

## Supplementary material

Table S1 **qRT-PCR primers in this study**.

Table S2 **Annotation of ***GmWRKY*** genes**. The names *GmWRKY1-64* and *GmWRKY65-182* genes are given according to Zhou et al. (2008) and Bencke-Malato et al. (2014); *GmWRKY183-186* genes are given according to the chromosome order in this study.

Table S3 **Digital expression analysis of ***GmWRKY*** genes**.

Table S4 *****GmWRKY*** genes differentially expressed under dehydration stress at 1, 6, and 12 h**.

Table S5 *****GmWRKY*** genes differentially expressed under salt stress at 1, 6, and 12 h**.

Table S6 **Optimal codons analysis in ***GmWRKY*** genes**. Number of codons in high bias dataset 2265; Number of codons in low bias dataset 3255; High bias was assigned to the dataset with the lower average NEC (effective number of codons); RSCU: relative synonymous codon usage; Codon UUC (Phe) chi value was 37.913; Codon UCC (Ser) chi value was 112.912; Codon UAC (Tyr) chi value was 19.766; Codon UGC (Cys) chi value was 5.112; Codon UCG (Ser) chi value was 22.781; Codon CUC (Leu) chi value was 83.801; Codon CCC (Pro) chi value was 7.139; Codon CAC (His) chi value was 60.096; Codon CGC (Arg) chi value was 17.807; Codon CCG (Pro) chi value was 61.074; Codon CGG (Arg) chi value was 13.621; Codon AUC (Ile) chi value was 9.526; Codon ACC (Thr) chi value was 28.927; Codon AAC (Asn) chi value was 43.255; Codon ACG (Thr) chi value was 23.339; Codon GUC (Val) chi value was 10.427; Codon GCC (Ala) chi value was 41.279; Codon GAC (Asp) chi value was 38.179; Codon GGC (Gly) chi value was 27.092; Codon GUG (Val) chi value was 19.760; Codon GCG (Ala) chi value was 39.804; Codon GAG (Glu) chi value was 13.304; Codon GGG (Gly) chi value was 10.177.

Table S7 *****cis***-element in differentially expressed genes of dehydration**.

Table S8 *****cis***-element in differentially expressed genes of salt**.

Figure S1 **Phylogenetic tree of AtWRKY and GmWRKY domain**. The phylogenetic tree was constructed using MAGE 6.0 by the Neighbor-Joining (NJ) method with 1000 bootstrap replicates.
