# Supplementary figures and images for: Global Analysis of WRKY Genes and Their Response to Dehydration and Salt Stress in Soybean
Source: Front Plant Sci. 2016 Feb 1;7:9. doi: 10.3389/fpls.2016.00009 (PMC4740950; doi:10.3389/fpls.2016.00009)

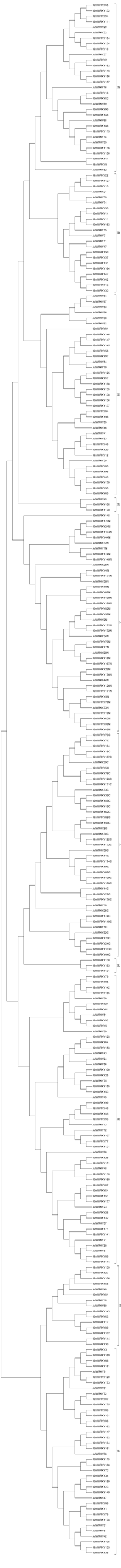

Supplement: Figure S1 — Phylogenetic tree of AtWRKY and GmWRKY domain. The phylogenetic tree was constructed using MAGE 6.0 by the Neighbor-Joining (NJ) method with 1000 bootstrap replicates. [file Image1.PDF]
